# Antidepressant Active Components of Bupleurum chinense DC-Paeonia lactiflora Pall Herb Pair: Pharmacological Mechanisms

**DOI:** 10.1155/2022/1024693

**Published:** 2022-11-09

**Authors:** Shimeng Lv, Yifan Zhao, Le Wang, Yihong Yu, Jiaxin Li, Yufei Huang, Wenhua Xu, Geqin Sun, Weibo Dai, Tingting Zhao, Dezhong Bi, Yuexiang Ma, Peng Sun

**Affiliations:** ^1^College of Traditional Chinese Medicine, Shandong University of Traditional Chinese Medicine, Jinan 250355, China; ^2^School of Pharmacy, Shandong University of Traditional Chinese Medicine, Jinan 250355, China; ^3^School of Management, Shandong University of Traditional Chinese Medicine, Jinan 250355, China; ^4^Department of Radiology, Ruijin Hospital, School of Medicine, Shanghai Jiao Tong University, Shanghai 200000, China; ^5^Preventive Treatment Center, Shenzhen Integrated Traditional Chinese and Western Medicine Hospital, Shenzhen 518027, China; ^6^Zhongshan Torch Development Zone People's Hospital, Zhongshan 528400, China; ^7^Department of Pharmacy, Zhongshan Hospital of Traditional Chinese Medicine, Zhongshan 528400, China; ^8^School of Foreign Language, Shandong University of Traditional Chinese Medicine, Jinan 250355, China; ^9^Experimental Center, Shandong University of Traditional Chinese Medicine, Jinan 250355, China; ^10^Innovation Institute of Chinese Medicine and Pharmacy, Shandong University of Traditional Chinese Medicine, Jinan 250355, China

## Abstract

Depression is a serious psychological disorder with a rapidly increasing incidence in recent years. Clinically, selective serotonin reuptake inhibitors are the main therapy. These drugs, have serious adverse reactions, however. Traditional Chinese medicine has the characteristics of multiple components, targets, and pathways, which has huge potential advantages for the treatment of depression. The antidepressant potential of the herbal combination of Bupleurum chinense DC (Chaihu) and Paeonia lactiflora Pall (Baishao) has been extensively studied previously. In this review, we summarized the antidepressant active components and mechanism of Chaihu-Baishao herb pair. We found that it works mainly through relieving oxidative stress, regulating HPA axis, and protecting neurons. Nevertheless, current research of this combined preparation still faces many challenges. On one hand, most of the current studies only stay at the level of animal models, lacking of sufficient clinical double-blind controlled trials for further verification. In addition, studies on the synergistic effect between different targets and signaling pathways are scarce. On the other hand, this preparation has numerous defects such as poor stability, low solubility, and difficulty in crossing the blood-brain barrier.

## 1. Introduction

Major depressive disorder, also known as depression, is a chronic, recurrent, and potentially life-threatening severe mental disorder [1]. According to the World Health Organization, depression is the main cause of disability in the world; more than 350 million people worldwide are suffering from depression, which increases the risk of death at any age, reduces the quality of life of depressed patients, and creates a burden on families and society. Clinically, Western medicine treatments such as the use of selective serotonin reuptake inhibitors are the main treatment for depression, but most of these compounds have issues that include delayed effects, high nonresponse rates, nausea, headaches, chronic sexual dysfunction, and weight gain [1–3].Therefore, it is important to develop more beneficial drugs for the treatment of depression. Traditional Chinese medicine (TCM) has great potential in the treatment of depression because of its multiple components that can act on multiple targets and pathways [4]. Some Chinese medicine formulas have significant effects on the treatment of depression with low toxicity and little to no side effects [5]. Therefore, in order to avoid the side effects and adverse reactions caused by Western medical treatments, scientific researchers have turned to TCM and its active ingredients as a way to treat depression [1].

Bupleurum chinense DC (Chaihu) is made from the dried roots of the Bupleurum plant. It is an herbal medicine that regulates and relieves liver qi. Modern research has found that Bupleurum chinense and its active ingredients have immunomodulatory, antiviral, hepatoprotective, antipyretic, and other pharmacological effects [6]. Paeonia lactiflora Pall (Baishao) is a traditional herb with a bitter, sour, and slightly cold taste. It can be used alone or as part of a drug combination. Modern pharmacological research shows that Baishao has anti-inflammatory, antiviral, antioxidant, and immunoregulatory properties as well as other functions [7]. The Chaihu-Baishao herbal combination is used in many antidepressant TCM compounds, such as Xiaoyao San, Chaihu Shugan San, and Sini San [8]. In this review, the research on the treatment of depression with the main active ingredients of the Chaihu-Baishao herb pair is summarized to provide a reference for future basic research and clinical applications.

## 2. Antidepressant mMchanisms of Chaihu-Baishao

### 2.1. Active Compounds in Chaihu-Baishao Inhibit Inflammation and Relieve Oxidative Stress

There is a large amount of evidence supporting the link between depression and inflammatory processes. It has been shown that inflammation increases an individual's susceptibility to depression. There is an increase in proinflammatory markers in patients with depression, and the use of proinflammatory drugs can increase the risk of depression [9]. Studies have found that oxidative stress may play an important role in the pathophysiology of depression. Patients with depression have elevated levels of malondialdehyde and the antioxidant enzymes, superoxide dismutase, and catalase, along with decreased levels of glutathione peroxidase [10]. Some scholars have found that 8-hydroxydeoxyglucose and F2 isoprostaglandin are found in patients with depression through meta-analysis. It is also believed that depression is accompanied by increased oxidative damage [11]. There is much evidence that inflammation-related diseases can be treated with various traditional medicines containing a variety of active natural compounds [12]. When antidepressants are used, the levels of peripheral inflammatory cytokines in patients with depression are reduced [13]. Therefore, inhibiting inflammation and relieving oxidative stress are important for the treatment of depression. Several of the previously studied active compounds of traditional medicines are reviewed below.

Saikosaponin D is a triterpene saponin compound extracted from Chaihu and has various pharmacological effects such as counteracting inflammation [14] and oxidative stress [15]. Tumor necrosis factor-*α* (TNF-*α*), interleukin-1*β* (IL-1*β*), and interleukin-6 (IL-6) are proinflammatory cytokines that regulate oxidative stress, apoptosis, and metabolism and cause damage to the branching process of neurons, which will in turn affect neuronal function [1]. Microglia's are the innate immune cells of the central nervous system and play a vital role in the process of neuroinflammation; they are found in abundance in the cerebrospinal fluid of depressed patients. A series of proinflammatory factors released by activated microglia were detected. In a lipopolysaccharide (LPS)-induced depression model, Su et al. gave mice an intraperitoneal injection of Saikosaponin D and found that it can improve the depression-like behavior of the mice and inhibit both the overexpression of the proinflammatory cytokines, TNF-*α*, IL-6, and 1 L-1*β*, and the activation of microglia induced by LPS in the mouse hippocampus [16]. The observed anti-inflammatory effect was also correlated with the inhibition of the high mobility group protein 1/Toll-like receptor 4 (TLR4)/nuclear transcription factor-*κ*B (NF-*κ*B) signaling pathway. The NF-*κ*B pathway is a typical inflammatory pathway, which can regulate the production of proinflammatory cytokines, leukocyte recruitment, or cell survival and cause the body to produce an inflammatory response [17]. It has been reported that Saponin D can downregulate the expression of NF-*κ*B in rat hippocampal neurons and improve the depression-like behavior induced by chronic unpredictable mild stress (CUMS) by downregulating Mir-155 expression and upregulating fibroblast growth factor 2 (FGF2) expression [18]. Quercetin is a flavonoid compound widely found in fruits and vegetables that has anti-inflammatory, antioxidant, antiviral, and anticancer effects [19]. It has been reported that quercetin can reverse the increase in lipid hydroperoxide content, induced by olfactory bulbectomy, in the hippocampus and improve the depression-like behavior of mice via a mechanism that is correlated with the enhancement of N-methyl-D-aspartic acid receptor expression [20].

**Figure 1:**
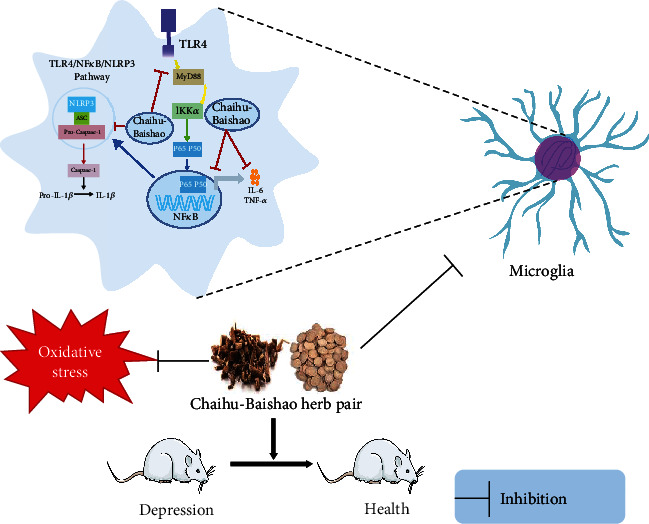
Active compounds in Chaihu-Baishao inhibit inflammation and relieve oxidative stress. TLR4: Toll-like receptor 4; NLRP3: nucleotide-binding oligomerization domain-like receptor protein 3; NF-*κ*B: nuclear transcription factor-*κ*B; TNF-*α*: Tumor necrosis factor-*α*; IL-6: interleukin-6; IL-1*β*: interleukin-1*β*.

Kaempferol is the main component of a variety of fruits and vegetables. Using a mouse depression model based on chronic social defeat stress, Gao et al. found that after intraperitoneal injection of kaempferol, the inflammatory response and oxidative stress in the prefrontal cortex of these mice was alleviated [21]. Kaempferol was also found to increase the activity of P-serine/threonine protein kinase (AKT) and *β*-catenin, but after using PI3-K inhibitors, the overall protective effect mediated by kaempferol was partially inhibited, indicating that kaempferol can enhance the antioxidant capacity and anti-inflammatory effects by enhancing the activity of the AKT/*β*-catenin cascade, thereby treating depression [21]. Additionally, caffeic acid, a catechol compound, is widely distributed in fruits, tea, and wine. In the LPS-induced depression model in mice, caffeic acid was found to reverse both the reduction of brain glutathione levels and the increase of malondialdehyde and proinflammatory cytokines [22]. Ferulic acid is a phenolic compound widely found in a variety of herbal medicines. There is evidence that in mouse models of cortisol (CORT)-induced depression, ferulic acid can improve the behavioral performance of depressed mice and simultaneously reduce malondialdehyde, nitrite, and protein carbonylation levels in the brain and increase nonprotein sulfhydryl levels [23]. Liu et al. found that administration of ferulic acid can reverse the CUMS-induced upregulation of the proinflammatory cytokines, IL-1*β*, IL-6, and TNF-*α*, in the prefrontal cortex of mice and the activation of microglia, NF-*κ*B signaling, and the nucleotide-binding oligomerization domain-like receptor protein 3 (NLRP3) inflammasome [24]. Gallic acid is a secondary plant metabolite, which is commonly found in many plant-based foods and beverages and has antioxidant activity. In a mouse model of poststroke depression (PSD), Nabavi et al. found that oxidative stress is closely related to the pathological process of stroke and PSD, and gallic acid can exert an antidepressant effect by inhibiting oxidative stress [25]. Paeoniflorin is one of the main biologically active ingredients extracted from the root of Paeonia lactiflora, which has antioxidation, anti-inflammatory, and other pharmacological effects [26]. Gasdermin D (GSDMD) is a member of the Gasdermin conserved protein family. When cell pyrolysis occurs, Caspase-1 is activated, which directly leads to GSDMD cleavage. GSDMD is cleaved out of the C-terminal and N-terminal domains that binds to the phospholipid protein on the cell membrane, resulting in the formation of cell membrane holes that cause cell rupture, cell content outflow, and a massive release of inflammatory factors. GSDMD can also activate Caspase-1 activation induced by NLRP3 [27]. Reports have shown that paeoniflorin can inhibit the expression of GSDMD, caspase-11, Caspase-1, NLRP3, IL-1*β*, and other proteins involved in pyroptosis signal transduction in microglia, as well as reduce inflammation and relieve symptoms of depression [28]. FGF2 is a neurotrophic and anti-inflammatory factor involved in regulating the proliferation, differentiation, and apoptosis of neurons in the brain. In a mouse depression model induced by LPS, Cheng et al. found that paeoniflorin can inhibit LPS-induced TLR4/NF-*κ*B/NLRP3 signaling in the hippocampus of mice, reduce the level of proinflammatory cytokines and microglia activation, and at the same time, increase neuronal FGF2 levels and dendritic spine density [29]. Neuropathic pain is a clinical problem that causes comorbid pain and depression. In a neuropathic pain mouse model, Bai et al. found that paeoniflorin can significantly improve the hyperalgesia and depression-like behavior of mice, reduce the level of proinflammatory cytokines, inhibit the excessive activation of microglia, and reduce the pathological damage of hippocampal cells in a way that is similar to the inhibition of the expression of TLR4/NF-*κ*B pathway related proteins [30]. Interferon-*α* (IFN-*α*) is a pleiotropic cytokine with antiviral and antiproliferative effects. It is widely used in cancer. However, studies have found that about 30%–50% of patients have symptoms of depression after receiving IFN-*α* treatment. Li et al. found that administration of paeoniflorin can improve the IFN-*α*-induced depression-like behavior in mice, while reducing inflammation levels in the serum, medial prefrontal cortex, ventral hippocampus, and amygdala [31]. Systemic lupus erythematosus is a chronic inflammatory autoimmune disease, and depression is one of its common complications. It was found that administration of paeoniflorin can inhibit the activity of the high-mobility group box 1 protein/TLR4/NF-*κ*B pathway and alleviate the level of inflammation in the serum and hippocampus, thereby treating lupus-induced depression [32]. Through component identification, Li found that Chaihu-Baishao mainly contain saikosaponin A, saikosaponin D, saikosaponin C, saikosaponin B2, paeoniflorin, albiflorin, and oxypaeoniflorin, and then combined with network pharmacology and metabolomics. Li found that Chaihu-Baishao plays an antidepressant effect by regulating arachidonic acid metabolism, the expression of Prostaglandin G/H synthase 1(PTGS1) and Prostaglandin G/H synthase 2(PTGS2) targets [8]. He et al. analyzed the changes of chemical constituents before and after the combination of Chaihu-Baishao by UPLC-MS background subtraction and metabolomics, founding that saikosaponin A, 3′-O-acetylation of saikosaponin A, and 4′-O-acetylation of saikosaponin A decreased after compatibility with Chaihu, and paeoniflorin, gallic acidpaeoniflorin or their isomers increased [33]. Sini Powder is a commonly used traditional Chinese medicine. In the prescription, Chaihu plays a major role, and Baishao plays an auxiliary role. It has the effect of soothing the liver and regulating the spleen [34]. It has been reported that Sini Powder can improve depression-like behavior and neuronal pathological damage in the hippocampus in CUMS rats, and its mechanism may be related to the inflammatory response mediated by the NLRP3 inflammasome signaling pathway [35]. In clinical studies, Sini Powder can effectively improve depression in cerebral infarction, type 2 diabetes and functional dyspepsia, and improve clinical efficacy [36–38] (Figure 1 and Table 1).

### 2.2. Active Compounds in Chaihu-Baishao Regulate the Hypothalamic-Pituitary-Adrenal (HPA) Axis

The HPA axis is an important part of the neuroendocrine system and helps control the response to stress. When the HPA axis is activated, the paraventricular nucleus of the hypothalamus releases corticotropin-releasing hormone (CRH), which sends a signal to the anterior pituitary gland to secrete adrenocorticotropic hormone (ACTH) into the bloodstream, and in turn, ACTH acts on the adrenal cortex to stimulate the secretion of CORT [39]. HPA axis hyperfunction is an important factor in the onset of depression as evidenced by increases in corticotropin-releasing hormone, ACTH, and glucocorticoid, an imbalance in the HPA axis negative feedback, an enlargement of the pituitary and adrenal glands, and the onset of hypercortisolemia that are seen in some depression patients [40].

**Figure 2:**
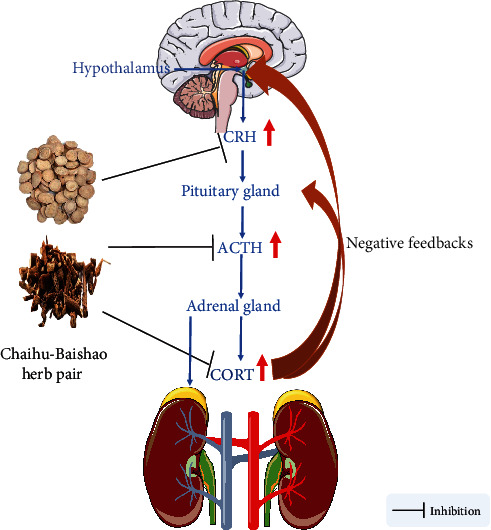
Active compounds in Chaihu-Baishao regulate the HPA axis. HPA: hypothalamic-pituitary-adrenal; CORT: cortisol; ACTH: adrenocorticotropic; CRH: corticotropin-releasing.

Saikosaponin A, a triterpene saponin extracted from Bupleurum, has anti-inflammatory and antioxidant effects [41]. In the perimenopausal depression model of female rats induced by CUMS, Saikosaponin A can improve the behavioral performance of these rats and reduce CRH mRNA, CRH protein, and serum CORT levels in the rat hypothalamus, while inhibiting the overexpression of hippocampal proinflammatory cytokines induced by CUMS [42]. Li et al. found that Saikosaponin D promotes hippocampal neurogenesis, alleviates the weight loss of rats induced by CUMS, and increases the sucrose consumption of rats and the movement distance in the open field test (OFT). They also found that it reduces the immobility time in the forced swimming test (FST), serum CORT levels, the inhibition of glucocorticoid receptor expression, and nuclear translocation induced by CUMS [43]. Adriamycin is a commonly used antitumor drug that has many adverse reactions. A previous study exploring the effect of quercetin on anxiety and depression-like behavior caused by Adriamycin showed that quercetin can improve the anxiety and depression-like behavior of rats, reduce serum CORT levels, inhibit the hyperactivity of the HPA axis, relieve brain oxidative damage, and regulate rat immune function [44]. Studies have also found that the combination of ferulic acid and levetiracetam improves epilepsy complicated by depression, restores serum CORT levels, and reduces the activity of proinflammatory cytokines and indoleamine 2,3-dioxygenase in the mouse brain [45]. Prenatal stress (PS) can increase the generation of depression, anxiety, attention deficit hyperactivity disorder, and other negative emotions and behaviors in offspring. After giving ferulic acid to rats in a prenatal stress model, Zheng et al. found that ferulic acid can exert an antidepressant effect by inhibiting the HPA axis activity and hippocampal inflammation in offspring rats [46]. Similarly, paeoniflorin can treat prenatal stress-induced depression-like behavior in rat offspring by promoting the nuclear translocation of glucocorticoid receptors, inhibiting the expression of a series of proteins and the formation of complexes (such as SNAP25), and inhibiting stress-induced HPA axis hyperactivity [47]. In FST-induced depression in rats, paeoniflorin can alleviate both the hyperactivity of the HPA axis and oxidative damage, increase plasma and hippocampal monoamine neurotransmitters and brain-derived neurotrophic factor (BDNF) levels, and promote gastrointestinal movement [48]. In a rat model of menopausal depression induced by CUMS combined with ovariectomy, Huang et al. found that paeoniflorin can improve the depression-like behavior of rats, while inhibiting both the overactivity of the HPA axis and the overexpression of the serotonin (5-HT_2A_) receptor and upregulating the expression of the 5-HT_1A_ receptor in the brain [49]. Protocatechuic acid ethyl ester (PCA) is a phenolic compound with neuroprotective effects. It has been reported that acute restraint stress can induce depression-like behavior in mice through neuronal oxidative damage, upregulation of HPA axis activity, and increases in serum CORT levels, all of which were reversed after PCA was administered [50] (Figure 2 and Table 2).

### 2.3. Active Compounds in Chaihu-Baishao Regulate Monoamine Neurotransmitters

Monoamine neurotransmitters are central neurotransmitters that are mainly catecholamines, such as dopamine, norepinephrine, and epinephrine, and indoleamines, such as 5-HT. Dopamine (DA) is an important regulator of learning and motivation [51]. 5-HT and norepinephrine are mainly involved in the regulation of emotional cognition and sleep, and when monoamine neurotransmitters are disordered, they can cause various emotional changes [40]. The classic monoamine hypothesis in depression predicts that the underlying pathophysiological basis of depression is the depletion of monoamine neurotransmitters in the central nervous system. The serum levels of monoamine neurotransmitters and their metabolites can be used as important biomarkers for the diagnosis of depression, and drugs that increase the synaptic concentration of monoamines can improve the symptoms of depression [1].

**Figure 3:**
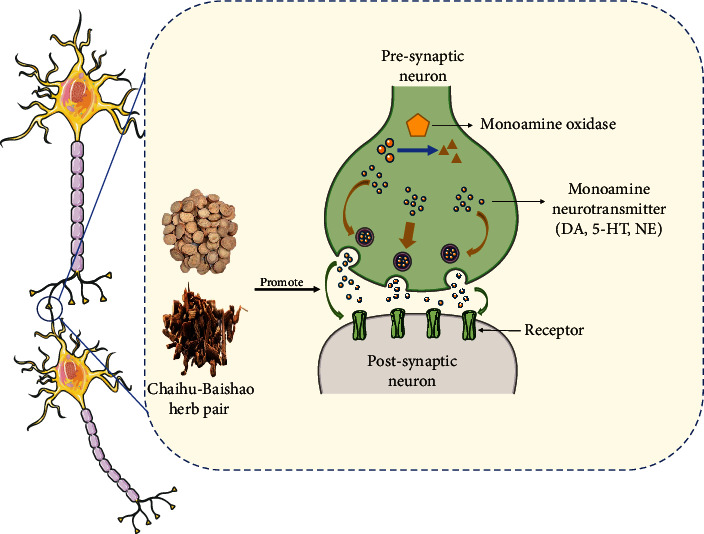
Active compounds in Chaihu-Baishao regulate monoamine neurotransmitters. 5-HT: serotonin; DA: dopamine; NE: norepinephrine.

It has been reported that Saikosaponin A can improve CUMS-induced depression-like behaviors in rats, and its antidepressant effect is believed to be related to the increase of dopamine content in the hippocampus of rats and the upregulation of hippocampal Proline-rich transmembrane protein 2 expression [52]. Similarly, in the same animal model of depression, Khan et al. found that quercetin can improve the behavioral performance of depressed mice, increase brain 5-HT levels, alleviate CUMS-induced brain inflammation and oxidative damage, and reduce brain glutamate levels [53]. There is evidence that in restraint stress-induced depression and anxiety models in mice, quercetin can treat anxiety and depression by regulating 5-HT and cholinergic neurotransmission and antioxidative damage as well as enhancing memory after restraint stress [54]. Acetylcholinesterase has the effect of terminating cholinergic neurotransmission [55]. Arsenic is an element that naturally exists in food, soil, and water. Exposure to arsenic can cause memory disorders, anxiety, depression, and other neurological perturbations and diseases. Samad et al. found that gallic acid can reverse the excessive increase in acetylcholinesterase activity induced by arsenic, alleviate the brain oxidative stress damage caused by arsenic, and protect memory function [56]. Additionally, gallic acid can also exert an antidepressant effect by increasing 5-HT and catecholamine levels in synaptic clefts in the central nervous system [57]. There are also reports showing that in olfactory bulbectomy-induced animal depression models, PCA can shorten the immobility time of rats in the OFT, increase the distance explored in the OFT, increase rat hippocampal monoamine neurotransmitters (5-HT, DA, and norepinephrine) and BDNF levels, reduce hippocampal CORT levels, and alleviate hippocampal neuroinflammation and oxidative damage [58]. It has been reported that Chaihu-Baishao can effectively improve postoperative depression and diarrhea in patients with colorectal cancer by regulating the imbalance of monoamine neurotransmitters (DA and 5-HT) [59] (Figure 3 and Table 3).

### 2.4. Active Compounds in Chaihu-Baishao Promote Hippocampal Neurogenesis and Regulate BDNF Levels

BDNF is a widely studied growth factor that plays an important role in neuronal maturation, synapse formation, and synaptic plasticity in the brain [60]. The “neurotrophic theory” posits that neurons will lose access to trophic factors as the expression level of BDNF decreases, leading to neuronal atrophy, decreased synaptic plasticity, and the onset of depression [61]. The optimization of BDNF levels helps synaptic plasticity and remodeling, improves neuronal damage, and relieves depression [62]. There is evidence that increasing the expression of BDNF in hippocampal astrocytes can treat depression and anxiety and stimulate hippocampal neurogenesis [63]. Adult hippocampal neurogenesis involves the proliferation, survival, differentiation, and integration of newborn neurons into preexisting neuronal networks [64]. There is evidence that hippocampal volume, the number of granular cells in the anterior and middle dentate gyrus, and the volume of the granular cell layer decrease in patients with depression [65], whereas increasing adult hippocampal neurogenesis can improve depression and anxiety [66].

Depression is also one of the common complications after stroke, though this pathogenesis has not been fully elucidated, and there is a lack of clinically effective treatments for stroke-induced depression. The cAMP response element-binding protein (CREB) is a protein that regulates gene transcription and acts as a chief transcription factor in the regulation of genes that encode proteins, such as BDNF, that are involved in synaptic plasticity and memory [67]. Studies have found that Saikosaponin A can significantly improve the depression-like behavior of PSD model rats, inhibit the apoptosis of the hippocampal meridian, and increase the levels of phosphorylated CREB and BDNF in the hippocampus [68]. Neuronal cell death occurs extensively during development and pathology, where it is especially important because of the limited capacity of adult neurons to proliferate or be replaced [69]. There are at least three known types of neuronal death, namely apoptosis, autophagy, and necrosis, and there is evidence that apoptosis is closely related to bipolar disorder (including depression) [70]. Inhibiting neuronal apoptosis can improve depression-like behavior [71, 72]. In LPS-induced depression in mice, Saikosaponin D can inhibit hippocampal neuronal apoptosis and inflammation through the lysophosphatidic acid 1/Ras homologous family member A (RhoA)/Rho protein kinase 2 pathway [73]. Rutin is a flavonoid, which is abundantly present in a variety of fruits and vegetables. It functions in cardiovascular protection and as an antiviral agent. In a recent study, rutin was shown to improve the CUMS-induced weight loss in mice and protect mouse hippocampal neurons [74]. Activation of tyrosine kinase B (TrkB) promotes neuronal survival, differentiation, and synapse formation [75].

**Figure 4:**
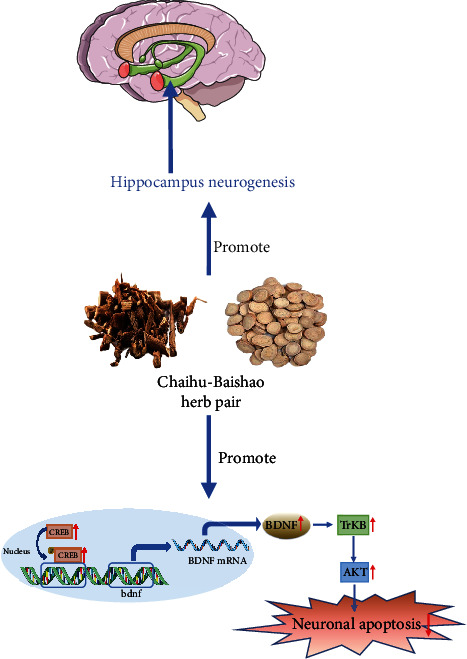
Active compounds in Chaihu-Baishao promote hippocampal neurogenesis and regulate BDNF levels. BDNF: brain-derived neurotrophic factor; AKT: serine/threonine protein kinase; TrKB: tyrosine kinase B; CREB: cAMP response element-binding protein.

Estrogen receptor alpha (ER*α*) is the main regulator mediated by estrogen and plays an important role in preventing depression and cardiovascular diseases. It has been reported that knocking out ER*α* induces depression in mice and reduces hippocampal BDNF and the phosphorylation of its downstream targets TrkB, AKT, and extracellular regulatory protein kinase 1/2 (ERK1/2). Quercetin can reverse the above phenomenon, alleviate cell apoptosis, and reverse the depression-like symptoms induced by ER*α* knockout [76]. Similarly, quercetin can also exert an antidepressant effect by regulating the levels of BDNF in the hippocampus and prefrontal cortex, as well as the levels of the related Copine 6 and the triggering receptor expressed on myeloid cells (TREM)-1 and TREM-2 [77]. Quercetin combined with exercise therapy can increase the expression of BDNF protein in 1,2-dimethylhydrazine-induced depression model rats with colorectal cancer and can act on the TrK*β*/*β*-chain protein axis to treat depression [78]. Liu et al. found that in the mouse depression model induced by CUMS, ferulic acid can significantly improve the behavioral performance in both the sucrose performance test and FST, upregulate BDNF and synapsin I levels in the prefrontal cortex and hippocampus, and increase hippocampal PSD95 protein expression [79]. In a recent report, paeoniflorin can improve the depression-like behavior of PSD model mice and increase the expression of BDNF and phosphorylated CREB in the CA1 region of the hippocampus [80]. Zhong et al. found that paeoniflorin can protect nerves by upregulating the activity of the ERK-CREB signaling pathway and treat CUMS-induced depression-like behavior in rats [81]. The alteration of synaptic plasticity, and specifically hippocampal long-term potentiation (LTP), also plays a role in the onset of depression [82]. There is evidence that in CUMS-induced animal depression models, hippocampal LTP is impaired, and Liu et al. found that administration of paeoniflorin can alleviate LTP injury in the hippocampal CAI area and increase both the density of hippocampal dendritic spines and the expression levels of BDNF and PSD95 [83]. There are also reports that paeoniflorin can enhance the expression and gene transcription of BDNF, activate the expression of TrkB, and promote proliferation and differentiation into astrocytes and the neurogenesis of neural stem cells in the rat hippocampal dentate gyrus [84] (Figure 4 and Table 4).

## 3. Discussion

Depression is a common neuropsychiatric disorder. Its symptoms and signs include lack of motivation, inability to feel happy, social withdrawal, cognitive difficulties, and changes in appetite. It belongs to the category of “mood disorder” in Chinese medicine [5]. In TCM theory, Chaihu can soothe the liver, and white peony root can restrain the qi. In the antidepressant Chinese medicine compounds, such as Xiaoyao Powder and Sini Powder, Chaihu and Baishao are often a “monarch and minister” collocation relationship [8]. In this review, we present the results of studies that have found that the active ingredients in Chaihu and Baishao that can function as an antidepressant through mechanisms that include reduction of inflammation and oxidative stress, neuronal protection, and the regulation of the HPA axis, neurotransmitters, and BDNF levels. However, the current research in these areas was only done at the cellular or animal level, and there have not been enough clinical trials to investigate whether they can effectively improve the symptoms of depressed patients. Furthermore, there are some active ingredients in Chaihu and Baishao, such as paeoniflorin, which are difficult to pass through the blood-brain barrier, thus limiting the efficacy. In recent years, the two-way signaling pathway between the brain and the gut microbiota has received much attention, and there is evidence that dysfunction of the gut-brain axis plays an important role in the pathogenesis of depression [85]. However, there is still a lack of sufficient research to show that the Chaihu-Baishao herb pair can treat depression by regulating the brain-gut axis. At the same time, most of the current research is limited to a single target or single signaling pathway, and the interaction mechanism between the target and the pathway lacks in-depth exploration. Clinically, the current main treatment methods are mainly western medicine, but there are serious adverse reactions. TCM has significant advantages in treatment of depression, which is worthy of clinical investigation. However, the current syndrome differentiation and classification of depression is complex and changeable, the standards are not uniform, and a complete diagnosis and treatment and efficacy evaluation system has not been formed. There are few case analyses in clinical research, and the quality is different.

In future research, therefore, a large number of randomized double-blind controlled trials are expected to be carried out in accordance with the standardization of syndrome differentiation to investigate whether Chaihu-Baishao can effectively improve depression. In order to apparently promote the efficacy of TCM, it is meaningful to strengthen the research on the targeted delivery system, prolonging action time, and increase concentration in central nervous system. Meanwhile, multiomics technology is beneficial for exploring the connection between targets or signaling pathways and the overall drug intervention mechanism, which could enrich the mechanism of action of TCM, providing guidance for industrial transformation and clinical application.

## Figures and Tables

**Table 1:** Active compounds in Bupleurum chinense DC-Paeonia lactiflora Pall herb pair inhibit inflammation and relieve oxidative stress.

Compound	Model	Animal species	Dosage	Behaviour involved	Mechanism of action/main indicators	References
Saikosaponin D	LPS	Male ICR mice	1 mg/kg	OFT, TST, FST, and SPT	Inhibition of microglia activation, the activity of TLR4/NF-*κ*B pathway HMGB1 translocation, and proinflammatory cytokines ↓	[16]

Quercetin	CUMS	Male SD rat	0.75, 1.5 mg/kg	OFT, TST, and FST, SPT	NF-*κ*B↓, FGF2↑, and miR-155↓	[18]
	OB	Female SWISS mice	10, 25, 50 mg/kg	FST, TST, OFT, and ST	LOOH↓	[20]

Kaempferol	CSDS	Male CD1 and C57 mice	10, 20 mg/kg	SPT, SIT, and TST	SOD↑, MDA↓, CAT↑, GPx↑, GST↑, P-AKT↑, and *β*-catenin↑	[21]

Caffeic acid	LPS	Male Swiss albino mice	30 mg/kg	OFT, FST, and TST	IL-6↓, MDA↓, GSH↑, and TNF-*α*↓	[22]

Ferulic acid	CORT	Male Swiss mice	1 mg/kg	TST, OFT, and ST	MDA↓, nitrite↓, and protein carbonylation↓	[23]
	CUMS	Male ICR mice	20,40, 80 mg/kg	SPT and TST	IL-1*β*,I L-6, TNF-*α* mRNA↓, NF-*κ*B↓ P-NF-*κ*B/NF-*κ*B↓, and inhibition of PFC microglia activation	[24]

Gallic acid	BCCAO	Male balb/c mice	25, 50 mg/kg	TST and SPT	Antioxidant stress	[25]

Paeoniflorin	RESP/LSP/ATP	Male C57BL/6 Mice	10, 20, 40 mg/kg	OFT, TST, and FST	Dendritic spines in hippocampal CA1 region↑, NLRP3↓, CASP-11↓, CASP-1↓, GSDMD↓, IL-1*β*, and microglia activation ↓	[28]
	LPS	Male ICR mice	20, 40, 80 mg/kg	SPT and FST	FGF2↑, IL-6↓, TNF-*α*↓, TLR4↓, P-NF-*κ*B↓, NLRP3↓, Cox-2↓, and dendritic spine density in CA3 area of hippocampus ↑	[29]
	Cuff	SPF male Balb/c mice	50, 100 mg/kg	SPT, FST, and TST	Inflammation in hippocampal CA3 area ↓, IL-6↓, TNF-*α*↓, IL-1↓, microglia activation ↓, and TLR4/NF-*κ*B pathway ↓	[30]
	IFN-*α*	Male C57BL/6Jmice	10, 20, 40 mg/kg	SPT, OFT, TST, and FST	Serum, mPFC, vHi, and amygdala inflammation levels↓	[31]
	SLE	Wild type mice and MRL/MpJ-Faslpr/ 2 J (MRL/lpr) mice	20 mg/kg	SPT, TST, and FST	Activity of HMGB1/TLR4/NF-*κ*B pathway↓, serum, and hippocampus contents of TNF-*α*, IL-1*β*, and IL-6↓	[32]

Notes: OFT: open field test; TST: tail suspension test; FST: forced swimming test; SPT: sucrose preference test; TLR4: Toll-like receptor 4; NF-*κ*B: nuclear transcription factor-*κ*B; HMGB1: High mobility group box 1; FGF2: fibroblast growth factor 2; LOOH: lipid hydroperoxides; SOD: Superoxide dismutase; MDA: malondialdehyde; CAT: catalase; GST: Glutathione S-transferase; AKT: serine/threonine protein kinase; IL-6: interleukin-6; TNF-*α*: Tumor necrosis factor-*α*; NLRP3: nucleotide-binding oligomerization domain-like receptor protein 3; CASP-11: Caspase-11; CASP-1: Caspase-1; GSDMD: Gasdermin D; IL-1*β*: interleukin-1*β*; TLR4: Toll-like receptor 4; LPS: lipopolysaccharide; CUMS: chronic unpredictable mild stress; OB: olfactory bulbectomy; CSDS: Chronic social stress defeat; CORT: cortisol; BCCAO: brain ischemia-reperfusion; ATP: adenosine triphosphate; IFN-*α*: Interferon-*α*; SLE: Systemic lupus erythematosus.

**Table 2:** Active compounds in Bupleurum chinense DC-Paeonia lactiflora Pall herb pair regulate the HPA axis.

Compound	Model	Animal species	Dosage	Behaviour involved	Mechanism of action/main indicators	References
Saikosaponin A	CUMS	Female Wistar rat	25, 50, 100 mg/kg	SPT, FST, and NSFT	Hypothalamus CRH mRNA, CRH protein↓, hippocampal proinflammatory cytokine↓, and CORT↓	[42]

Saikosaponin D	CUMS	Male SD rat	0.75, 1.5 mg/kg	SPT, OFT, and FST	CORT↓, GR↑, weight ↑, p-CREB↑, BDNF↑, and promoting the generation of hippocampal neurons	[43]

Quercetin	Adriamycin	Male Wistar rat	60 mg/kg	FST, OFT, and EPM	CORT↓, relieving brain oxidative stress damage, and regulating immune function	[44]

Ferulic acid	Pentylenetetrazole kindling epilepsy	Male Swiss albino mice	40, 80 mg/kg	TST, SPT	CORT↓, TNF-*α*↓, and IL-1*β*↓	[45]
	PS	Female, male SD rats	12.5, 25, 50 mg/kg	SPT, OFT, and FST	Serum ACTH↓, CORT↓, hippocampus GR↑, inhibiting hippocampal inflammation, and improving neuronal damage in hippocampal CA3 area	[46]

Paeoniflorin	PS	Female, male SD rats	15, 30, 60 mg/kg	SPT, FST, and OFT	Serum CRH, ACTH, CORT↓, and relieving neuronal damage in hippocampal CA3 area	[47]
	FST	Male SD rat	10 mg/kg	FST and OFT	Promote gastrointestinal motility, plasma motilin↑, CRH↓, ACTH↓, CORT↓, BDNF↓, norepinephrine↑, and oxidative stress↓	[48]
	CUMS	SD female rats	45 mg/kg	OFT and SPT	HPA axis activity↓, brain 5-HT2AR↓, and brain 5-HT1AR↑	[49]

Protocatechuic acid	ARS	Male, female, Swiss albino mice	100, 200 mg/kg	FST and OFT	Serum CORT↓ and hippocampal oxidative stress↓,	[50]

**Note:** CUMS: chronic unpredictable mild stress; PS: Prenatal stress; FST: forced swimming test; ARS: Acute restraint stress; SPT: sucrose preference test; NSFT: novelty suppressed feeding test; OFT: open field test; EPM: Elevated Plus Maze; CRH: corticotropin-releasing hormone; GR: Glucocorticoid Receptor; BDNF: brain-derived neurotrophic factor; ACTH: adrenocorticotropic hormone; HPA: hypothalamic-pituitary-adrenal; 5-HT: serotonin; MDA: malondialdehyde; CORT: cortisol.

**Table 3:** Active compounds in Bupleurum chinense DC-Paeonia lactiflora Pall herb pair regulate monoamine neurotransmitters.

Compound	Model	Animal species	Dosage	Behaviour involved	Mechanism of action/main indicators	References
Saikosaponin A	CUMS	Male SD rat	50 mg/kg	OFT and SPT	Weight↑, hippocampus DA↑, and hippocampus PRRT2↑	[52]

Quercetin	CUMS	Male Swiss albino mice	25 mg/kg	MFST, TST, and OFT	Brain5-HT↑, brain glutamate, IL-6, TNF-*α*↓, and relieving brain oxidative stress	[53]
	Restraint stress	Male Albaino wistar mice	20 mg/kg/ml	FST, LDA, EPM, and MWM	Prevent oxidase damage, regulate 5-HT, and cholinergic	[54]

Gallic acid	iAS	SD male rat	50, 100 mg/kg	EPM, LDA, FST, and MWM	Relieve brain oxidative stress and brain AChE↓	[56]

Protocatechuic acid	OB	Male Wistar rat	100, 200 mg/kg	FST and OFT	Hippocampus 5-HT, norepinephrine, DA↑, hippocampus BNDF↑, hippocampus TNF-*α*, IL-6, CORT, and hippocampus oxidase damage↓	[58]

**Note:** LDA: Light-dark activity box; iAS: Arsenic; OB: olfactory bulbectomy; MWM: Morris water maze; FST: forced swimming test; EPM: Elevated Plus Maze; OFT: open field test; 5-HT: serotonin; CORT: cortisol; IL-6: interleukin-6; TNF-*α*: Tumor necrosis factor-*α*; AChE: acetylcholinesterase; DA: dopamine; BDNF: brain-derived neurotrophic factor; MFST: Modified Forced Swim Test; OB: olfactory bulbectomy; TST: tail suspension test; CUMS: chronic unpredictable mild stress.

**Table 4:** Active compounds in Bupleurum chinense DC-Paeonia lactiflora Pall herb pair promote hippocampal neurogenesis and regulate BDNF levels.

Compound	Model	Animal species	Dosage	Behaviour involved	Mechanism of action/ Main indicators	References
Saikosaponin A	MCAO+CUMS+isolation	Male SD rat	5 mg/kg	SPT, OFT, BWT, FST	Weight↑, hippocampus BDNF, P-CREB↑, inhibiting neuronal apoptosis, hippocampus Bax, caspase-3↓,	[68]

Saikosaponin D	LPS	Male ICR mice	0.5, 1 mg/kg	TST, OFT	Proinflammatory cytokines↓, inhibiting the activity of LPA1/RhoA/ROCK2 signaling pathway, relieving apoptosis of hippocampal neurons	[73]

Rutin	CUMS	Adult Swiss albino mice	100 mg/kg	OFT, SPT, EPM, NOR, BWT	Relieving hippocampal damage in CA3 area	[74]

Quercetin	ER*α* receptor knockout	Female C57bl/6 and ER*α*-KO mice	100 mg/kg	OFT, TST, FST	Number of hippocampal neurons↑, relieve hippocampal cell apoptosis, Hippocampus BDNF,P-TrKB,P-AKT,P-ERK1/2↑	[76]
	DMH	Male Wistar rat	50 mg/kg	OFT, FST	Proinflammatory cytokines↓, BDNF↑, number of neurons↑, TrKB↑, *β*-catenin↑, incidence of rectal cancer↓,	[78]

Ferulic acid	CUMS	Male ICR mice	20, 40 mg/kg	SPT, FST	PFC and hippocampus BDNF, Synapsin I↑, hippocampus PSD95↑	[79]

Paeoniflorin	MCAO+CUMS	Male SD rat	5 mg/kg	BBT, SPT, OFT	Weight↑, hippocampus CA1 area BDNF, P-CREB↑	[80]
	CUMS	Male SD rat	30, 60 mg/kg	SPT, FST, LAT	Number of neurons in hippocampal CA3 area↑, upregulate the ERK-CREB signaling pathway	[81]
	CUMS	C57BL/6 Wild-type male mice	20 mg/kg	SPT, FST, TST	Relieve hippocampal CA1 LTP damage, hippocampus dendritic spine density, BDNF, PSD95↑	[82]
	CUMS	Male SD rats	60 mg/kg	SPT	Promote neurogenesis in the hippocampus dentate gyrus	[84]

**Note:** CUMS: chronic unpredictable mild stress; MCAO: middle cerebral artery occlusion; SPT: sucrose preference test; OFT: open field test; FST: forced swimming test; BWT: Beam walking test; BDNF: brain-derived neurotrophic factor; CREB: cAMP response element-binding protein; LPS: lipopolysaccharide; LPA-1: specific cell surface G protein–coupled receptors; ROCK: Rho-kinase; NOR: Novel object recognition test; TST: tail suspension test; TrKB: tyrosine kinase B; AKT:serine/threonine protein kinase; ERK: extracellular regulatory protein kinase; ER*α*: Estrogen receptor alpha; DMH: 1,2-dimethylhydrazine; BBT: Beam balance test; LAT: Locomotor Activity Test; LTP: long-term potentiation.

## References

[1] Wang Y. S., Shen C. Y., Jiang J. G. (2019). Antidepressant active ingredients from herbs and nutraceuticals used in TCM: pharmacological mechanisms and prospects for drug discovery. *Pharmacological Research*.

[2] Qu S. Y., Li X. Y., Heng X. (2021). Analysis of antidepressant activity of Huang-Lian Jie-Du decoction through network pharmacology and metabolomics. *Frontiers in Pharmacology*.

[3] Wei Y., Chang L., Hashimoto K. (2022). Molecular mechanisms underlying the antidepressant actions of arketamine: beyond the NMDA receptor. *Molecular Psychiatry*.

[4] Zhang Y., Li X., Xu X., Yang N. (2019). Mechanisms of Paeonia lactiflora in treatment of ulcerative colitis: a network pharmacological study. *Medical Science Monitor*.

[5] Chi X., Wang S., Baloch Z. (2019). Research progress on classical traditional Chinese medicine formula lily bulb and Rehmannia decoction in the treatment of depression. *Biomedicine & Pharmacotherapy*.

[6] Law B. Y., Mo J. F., Wong V. K. (2014). Autophagic effects of Chaihu (dried roots of Bupleurum Chinense DC or Bupleurum scorzoneraefolium WILD). *Chinese Medicine*.

[7] Xue X., Liu G., Wei Y. (2021). Multi-element characteristics of Chinese medical Baishao (Paeoniae radix Alba) and their decoctions. *Biological Trace Element Research*.

[8] Li X., Qin X. M., Tian J. S., Gao X. X., Du G. H., Zhou Y. Z. (2021). Integrated network pharmacology and metabolomics to dissect the combination mechanisms of Bupleurum chinense DC -Paeonia lactiflora Pall herb pair for treating depression. *Journal of Ethnopharmacology*.

[9] Kohler O., Krogh J., Mors O., Benros M. E. (2016). Inflammation in depression and the potential for anti-inflammatory treatment. *Current Neuropharmacology*.

[10] Tsai M. C., Huang T. L. (2016). Increased activities of both superoxide dismutase and catalase were indicators of acute depressive episodes in patients with major depressive disorder. *Psychiatry Research*.

[11] Black C. N., Bot M., Scheffer P. G., Cuijpers P., Penninx B. W. (2015). Is depression associated with increased oxidative stress? A systematic review and meta-analysis. *A Systematic Review and Meta-Analysis. Psychoneuroendocrinology*.

[12] Zeng K.-W., Ming-Yao G. (2021). Annual advances of integrative pharmacology in 2020. *Traditional Medicine Research*.

[13] Liu J. J., Wei Y. B., Strawbridge R. (2020). Peripheral cytokine levels and response to antidepressant treatment in depression: a systematic review and meta-analysis. *Molecular Psychiatry*.

[14] Jiang J., Meng Y., Hu S., Botchway B. O. A., Zhang Y., Liu X. (2020). Saikosaponin d: a potential therapeutic drug for osteoarthritis. *Journal of Tissue Engineering and Regenerative Medicine*.

[15] Zhang B. Z., Guo X. T., Chen J. W. (2014). Saikosaponin-d attenuates heat stress-induced oxidative damage in LLC-PK1Cells by increasing the expression of anti-oxidant enzymes and HSP72. *The American Journal of Chinese Medicine*.

[16] Su J., Pan Y. W., Wang S. Q., Li X. Z., Huang F., Ma S. P. (2020). Saikosaponin-d attenuated lipopolysaccharide-induced depressive-like behaviors via inhibiting microglia activation and neuroinflammation. *International Immunopharmacology*.

[17] Lawrence T. (2009). The nuclear factor NF-kappa B pathway in inflammation. *Cold Spring Harbor Perspectives in Biology*.

[18] Chao B., Huang S., Pan J., Zhang Y., Wang Y. (2020). Saikosaponin d downregulates microRNA-155 and upregulates FGF2 to improve depression-like behaviors in rats induced by unpredictable chronic mild stress by negatively regulating NF-*κ*B. *Brain Research Bulletin*.

[19] Li Y., Yao J., Han C. (2016). Quercetin, inflammation and immunity. *Nutrients*.

[20] Holzmann I., da Silva L. M., Corrêa da Silva J. A., Steimbach V. M., de Souza M. M. (2015). Antidepressant-like effect of quercetin in bulbectomized mice and involvement of the antioxidant defenses, and the glutamatergic and oxidonitrergic pathways. *Pharmacology, Biochemistry, and Behavior*.

[21] Gao W., Wang W., Peng Y., Deng Z. (2019). Antidepressive effects of kaempferol mediated by reduction of oxidative stress, proinflammatory cytokines and up-regulation of AKT/*β*-catenin cascade. *Metabolic Brain Disease*.

[22] Basu Mallik S., Mudgal J., Nampoothiri M. (2016). Caffeic acid attenuates lipopolysaccharide-induced sickness behaviour and neuroinflammation in mice. *Neuroscience Letters*.

[23] Zeni A. L. B., Camargo A., Dalmagro A. P. (2017). Ferulic acid reverses depression-like behavior and oxidative stress induced by chronic corticosterone treatment in mice. *Steroids*.

[24] Liu Y. M., Shen J. D., Xu L. P., Li H. B., Li Y. C., Yi L. T. (2017). Ferulic acid inhibits neuro-inflammation in mice exposed to chronic unpredictable mild stress. *International Immunopharmacology*.

[25] Nabavi S. F., Habtemariam S., Di Lorenzo A. (2016). Post-stroke depression modulation and in vivo antioxidant activity of gallic acid and its synthetic derivatives in a murine model system. *Nutrients*.

[26] Zhou Y. X., Gong X. H., Zhang H., Peng C. (2020). A review on the pharmacokinetics of paeoniflorin and its anti-inflammatory and immunomodulatory effects. *Biomedicine & Pharmacotherapy*.

[27] Eldridge M. J. G., Sanchez-Garrido J., Hoben G. F., Goddard P. J., Shenoy A. R. (2017). The atypical ubiquitin E2 conjugase UBE2L3 is an indirect caspase-1 target and controls IL-1*β* secretion by inflammasomes. *Cell Reports*.

[28] Tian D. D., Wang M., Liu A. (2021). Antidepressant effect of paeoniflorin is through inhibiting pyroptosis CASP-11/GSDMD pathway. *Molecular Neurobiology*.

[29] Cheng J., Chen M., Wan H. Q. (2021). Paeoniflorin exerts antidepressant-like effects through enhancing neuronal FGF-2 by microglial inactivation. *Journal of Ethnopharmacology*.

[30] Bai H., Chen S., Yuan T., Xu D., Cui S., Li X. (2021). Paeoniflorin ameliorates neuropathic pain-induced depression-like behaviors in mice by inhibiting hippocampal neuroinflammation activated via TLR4/NF-*κ*B pathway. *Korean Journal of Physiology & Pharmacology*.

[31] Li J., Huang S., Huang W. (2017). Paeoniflorin ameliorates interferon-alpha-induced neuroinflammation and depressive-like behaviors in mice. *Oncotarget*.

[32] Wang S., Zhao X., Qiao Z., Jia X., Qi Y. (2018). Paeoniflorin attenuates depressive behaviors in systemic lupus erythematosus mice. *Biomedicine & Pharmacotherapy*.

[33] He J., Gao X. X., Tian J. S., Qin X. M., Du G. H., Zhou Y. Z. (2018). Changes of chemical composition of Bupleuri radix-Paeoniae radix Alba herb pair before and after compatibility by UPLC-MS background subtraction and metabonomics. *Zhong Cao Yao*.

[34] Wu H. W., Li D. H., Zhang Y. G. (2021). Research progress of Sini powder and prediction analysis on Q-markers. *Zhong Hua Zhong Yi Yao Xue.*.

[35] Wang W., Zhou Y. Y., Yu X. M., Jiang X. C., Tian D. Z., Xiao M. (2022). Effect of Sinisan on NLRP3 inflammasomes and depression-like behaviors in depressed rats. *Zhong Guo Shi Yan Fang Ji Xue Za Zhi*.

[36] Rao K. H., Jf X., Zhao L. Q., Huang C. H., Li D. H. (2021). Effects of Jiawei Sini powder on the levels of plasma IL-1*β*, IL-18, Hcy and neural factors in patients with depression after acute cerebral infarction. *Shi Zhen Guo Yi Guo Yao*.

[37] Zhang Q., Z Q, D ST, J B (2022). Clinical observation on Sini powder combined with Gan Mai Dazao decoction in the treatment of type 2 diabetes mellitus complicated with depression and anxiety. *Guang Zhou Zhong Yi Yao Da Xue Xue Bao*.

[38] Huang X. Y. (2022). Observation on the curative effect of Sini powder in the treatment of functional dyspepsia with depression. *Nei Meng Gu Zhong Yi Yao*.

[39] Frankiensztajn L. M., Elliott E., Koren O. (2020). The microbiota and the hypothalamus-pituitary-adrenocortical (HPA) axis, implications for anxiety and stress disorders. *Current Opinion in Neurobiology*.

[40] Wang X. L., Feng S. T., Wang Y. T., Chen N. H., Wang Z. Z., Zhang Y. (2021). Paeoniflorin: a neuroprotective monoterpenoid glycoside with promising anti- depressive properties. *Phytomedicine*.

[41] Zhou F., Wang N., Yang L., Zhang L. C., Meng L. J., Xia Y. C. (2019). Saikosaponin a protects against dextran sulfate sodium-induced colitis in mice. *International Immunopharmacology*.

[42] Chen X. Q., Chen S. J., Liang W. N. (2018). Saikosaponin a attenuates perimenopausal depression-like symptoms by chronic unpredictable mild stress. *Neuroscience Letters*.

[43] Li H. Y., Zhao Y. H., Zeng M. J. (2017). Saikosaponin D relieves unpredictable chronic mild stress induced depressive-like behavior in rats: involvement of HPA axis and hippocampal neurogenesis. *Psychopharmacology*.

[44] Merzoug S., Toumi M. L., Tahraoui A. (2014). Quercetin mitigates adriamycin-induced anxiety- and depression-like behaviors, immune dysfunction, and brain oxidative stress in rats. *Naunyn-Schmiedeberg's Archives of Pharmacology*.

[45] Singh T., Kaur T., Goel R. K. (2017). Ferulic acid supplementation for management of depression in epilepsy. *Neurochemical Research*.

[46] Zheng X., Cheng Y., Chen Y. (2019). Ferulic acid improves depressive-like behavior in prenatally-stressed offspring rats via anti-inflammatory activity and HPA Axis. *International Journal of Molecular Sciences*.

[47] Chun Li Y., Xing Zheng X., Zhe Xia S. (2020). Paeoniflorin ameliorates depressive-like behavior in prenatally stressed offspring by restoring the HPA axis- and glucocorticoid receptor- associated dysfunction. *Journal of Affective Disorders*.

[48] Mu D. Z., Xue M., Xu J. J. (2020). Antidepression and prokinetic effects of paeoniflorin on rats in the forced swimming test via polypharmacology. *Evidence-Based Complementary and Alternative Medicine*.

[49] Huang H., Zhao J., Jiang L. (2015). Paeoniflorin improves menopause depression in ovariectomized rats under chronic unpredictable mild stress. *International Journal of Clinical and Experimental Medicine*.

[50] Thakare V. N., Dhakane V. D., Patel B. M. (2017). Attenuation of acute restraint stress-induced depressive like behavior and hippocampal alterations with protocatechuic acid treatment in mice. *Metabolic Brain Disease*.

[51] Berke J. D. (2018). What does dopamine mean?. *Nature Neuroscience*.

[52] Guo J., Zhang F., Gao J. (2020). Proteomics-based screening of the target proteins associated with antidepressant-like effect and mechanism of saikosaponin a. *Journal of Cellular and Molecular Medicine*.

[53] Khan K., Najmi A. K., Akhtar M. (2019). A natural phenolic compound quercetin showed the usefulness by targeting inflammatory, oxidative stress markers and augment 5-HT levels in one of the animal models of depression in mice. *Drug Research*.

[54] Samad N., Saleem A., Yasmin F., Shehzad M. A. (2018). Quercetin protects against stress-induced anxiety- and depression-like behavior and improves memory in male mice. *Physiological Research*.

[55] Lane R. M., Kivipelto M., Greig N. H. (2004). Acetylcholinesterase and its inhibition in Alzheimer disease. *Clinical Neuropharmacology*.

[56] Samad N., Jabeen S., Imran I., Zulfiqar I., Bilal K. (2019). Protective effect of gallic acid against arsenic-induced anxiety-/depression- like behaviors and memory impairment in male rats. *Metabolic Brain Disease*.

[57] Can Ö. D., Turan N., Demir Özkay Ü., Öztürk Y. (2017). Antidepressant-like effect of gallic acid in mice: dual involvement of serotonergic and catecholaminergic systems. *Life Sciences*.

[58] Thakare V. N., Patil R. R., Suralkar A. A., Dhakane V. D., Patel B. M. (2019). Protocatechuic acid attenuate depressive-like behavior in olfactory bulbectomized rat model: behavioral and neurobiochemical investigations. *Metabolic Brain Disease*.

[59] Björkholm C., Monteggia L. M. (2016). BDNF - a key transducer of antidepressant effects. *Neuropharmacology*.

[60] van Zutphen E. M., Rhebergen D., van Exel E. (2019). Brain-derived neurotrophic factor as a possible predictor of electroconvulsive therapy outcome. *Psychiatry*.

[61] Phillips C. (2017). Brain-derived neurotrophic factor, depression, and physical activity: making the neuroplastic connection. *Neural Plasticity*.

[62] Mx H., Qq X., Hc Z. (2021). Dose-effect relationship of Chaihu and Baishao on improving depression and diarrhea in patients with colorectal cancer surgery. *Zhong Guo Zhong Xi Yi Jie He Wai Ke Za Zhi*.

[63] Quesseveur G., David D. J., Gaillard M. C. (2013). BDNF overexpression in mouse hippocampal astrocytes promotes local neurogenesis and elicits anxiolytic-like activities. *Psychiatry*.

[64] de Miranda A. S., Zhang C. J., Katsumoto A., Teixeira A. L. (2017). Hippocampal adult neurogenesis: does the immune system matter?. *Journal of the Neurological Sciences*.

[65] Mahar I., Bambico F. R., Mechawar N., Nobrega J. N. (2014). Stress, serotonin, and hippocampal neurogenesis in relation to depression and antidepressant effects. *Neuroscience and Biobehavioral Reviews*.

[66] Hill A. S., Sahay A., Hen R. (2015). Increasing adult hippocampal neurogenesis is sufficient to reduce anxiety and depression-like behaviors. *Neuropsychopharmacology*.

[67] Ghasemzadeh Z., Sardari M., Javadi P., Rezayof A. (2020). Expression analysis of hippocampal and amygdala CREB-BDNF signaling pathway in nicotine-induced reward under stress in rats. *Brain Research*.

[68] Wang A. R., Mi L. F., Zhang Z. L. (2021). Saikosaponin a improved depression-like behavior and inhibited hippocampal neuronal apoptosis after cerebral ischemia through p-CREB/BDNF pathway. *Behavioural Brain Research*.

[69] Fricker M., Tolkovsky A. M., Borutaite V., Coleman M., Brown G. C. (2018). Neuronal cell death. *Physiological Reviews*.

[70] Uribe E., Wix R. (2012). Neuronal migration, apoptosis and bipolar disorder. *Revista de Psiquiatría y Salud Mental*.

[71] Fan C., Song Q., Wang P., Li Y., Yang M., Yu S. Y. (2018). Neuroprotective effects of ginsenoside-Rg1 against depression-like behaviors via suppressing glial activation, synaptic deficits, and neuronal apoptosis in rats. *Frontiers in Immunology*.

[72] Fan C., Song Q., Wang P., Li Y., Yang M., Yu S. Y. (2019). Neuroprotective effects of curcumin on IL-1*β*-induced neuronal apoptosis and depression-like behaviors caused by chronic stress in rats. *Frontiers in Cellular Neuroscience*.

[73] Xu L., Su J., Guo L., Wang S., Deng X., Ma S. (2019). Modulation of LPA1 receptor-mediated neuronal apoptosis by Saikosaponin-d: a target involved in depression. *Neuropharmacology*.

[74] Parashar A., Mehta V., Udayabanu M. (2017). Rutin alleviates chronic unpredictable stress-induced behavioral alterations and hippocampal damage in mice. *Neuroscience Letters*.

[75] Peng Z., Zhang C., Yan L. (2020). EPA is more effective than DHA to improve depression-like behavior, glia cell dysfunction and hippocampal apoptosis signaling in a chronic stress-induced rat model of depression. *International Journal of Molecular Sciences*.

[76] Wang G., Li Y., Lei C. (2021). Quercetin exerts antidepressant and cardioprotective effects in estrogen receptor *α*-deficient female mice via BDNF-AKT/ERK1/2 signaling. *The Journal of Steroid Biochemistry and Molecular Biology*.

[77] Fang K., Li H. R., Chen X. X. (2020). Quercetin alleviates LPS-induced depression-like behavior in rats via regulating BDNF-related imbalance of Copine 6 and TREM1/2 in the hippocampus and PFC. *Frontiers in Pharmacology*.

[78] Sadighparvar S., Darband S. G., Yousefi B. (2020). Combination of quercetin and exercise training attenuates depression in rats with 1,2-dimethylhydrazine-induced colorectal cancer: possible involvement of inflammation and BDNF signalling. *Experimental Physiology*.

[79] Liu Y. M., Hu C. Y., Shen J. D., Wu S. H., Li Y. C., Yi L. T. (2017). Elevation of synaptic protein is associated with the antidepressant-like effects of ferulic acid in a chronic model of depression. *Physiology & Behavior*.

[80] Hu M. Z., Wang A. R., Zhao Z. Y., Chen X. Y., Li Y. B., Liu B. (2019). Antidepressant-like effects of paeoniflorin on post-stroke depression in a rat model. *Neurological Research*.

[81] Zhong X., Li G., Qiu F., Huang Z. (2018). Paeoniflorin ameliorates chronic stress-induced depression-like behaviors and neuronal damages in rats via activation of the ERK-CREB pathway. *Front Psychiatry*.

[82] Duman R. S., Aghajanian G. K., Sanacora G., Krystal J. H. (2016). Synaptic plasticity and depression: new insights from stress and rapid- acting antidepressants. *Nature Medicine*.

[83] Liu S. C., Hu W. Y., Zhang W. Y. (2019). Paeoniflorin attenuates impairment of spatial learning and hippocampal long-term potentiation in mice subjected to chronic unpredictable mild stress. *Psychopharmacology*.

[84] Chen L. B., Qiu F. M., Zhong X. M., Hong C., Huang Z. (2019). Promoting neurogenesis in hippocampal dentate gyrus of chronic unpredictable stress-induced depressive-like rats with paeoniflorin. *Journal of Integrative Neuroscience*.

[85] Du Y., Gao X. R., Peng L., Ge J. F. (2020). Crosstalk between the microbiota-gut-brain axis and depression. *Heliyon*.

